# Ligand design strategies to increase stability of gadolinium-based magnetic resonance imaging contrast agents

**DOI:** 10.1038/s41467-019-09342-3

**Published:** 2019-03-29

**Authors:** Thomas J. Clough, Lijun Jiang, Ka-Leung Wong, Nicholas J. Long

**Affiliations:** 1 Department of Chemistry, Imperial College London, Molecular Sciences Research Hub, White City Campus, Wood Lane London, W12 0BZ UK; 2 Department of Chemistry, Hong Kong Baptist University, Kowloon Tong Hong Kong SAR, China

## Abstract

Gadolinium(III) complexes have been widely utilised as magnetic resonance imaging (MRI) contrast agents for decades. In recent years however, concerns have developed about their toxicity, believed to derive from demetallation of the complexes in vivo, and the relatively large quantities of compound required for a successful scan. Recent efforts have sought to enhance the relaxivity of trivalent gadolinium complexes without sacrificing their stability. This review aims to examine the strategic design of ligands synthesised for this purpose, provide an overview of recent successes in gadolinium-based contrast agent development and assess the requirements for clinical translation.

## Abstract (web summary)

Gadolinium(III) complexes are strong enhancers of magnetic resonance imaging (MRI) signals, thus are widely used as contrast agents despite their potential toxicity. Here, the authors review ligand design approaches aimed at improving the stability of Gd(III)-based MRI contrast agents.

## Introduction

Skilful and strategic ligand design is key for any coordination chemist. Adapting and refining a ligand allows a captive metal’s potential to be unlocked and its capabilities to be fully exploited. If a metal complex is destined for use in a biological setting, it is the ligand motif which dictates the complex’s distribution, localisation and behaviour in a dynamic system. Recent history of gadolinium-based contrast agents (GBCAs) for magnetic resonance imaging (MRI) exemplifies the critical nature of ligand design in medical imaging. Contrast agents enhance the longitudinal (*T*_1_) or transverse (*T*_2_) relaxation rates of water molecules in their vicinity, resulting in greater contrast between different biological tissues, allowing them to be distinguished more readily^1–7^. Paramagnetic species are especially adept at enhancing *T*_1_ relaxation^7^. Gd^3+^ is an f-block metal ion with seven unpaired electrons, rendering it highly paramagnetic and a prime candidate for use in contrast agents^7,8^.

**Fig. 1:**
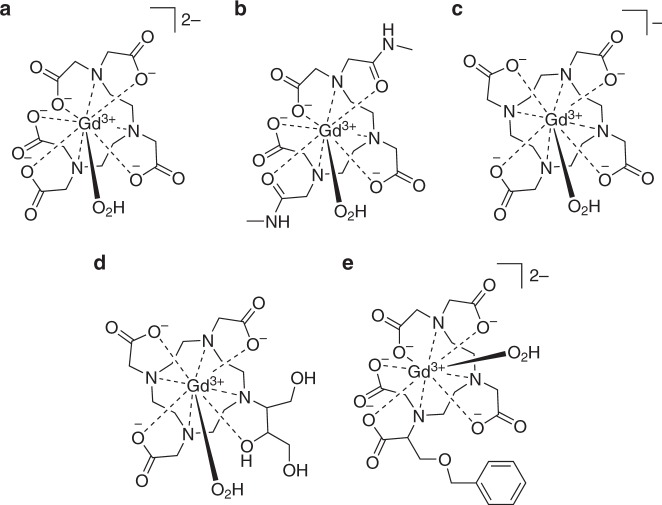
The five most commonly administered gadolinium-based contrast agents for clinical MRI^10^. **a** Magnevist^®^, [Gd(OH_2_)(dtpa)]^2−^. **b** Omniscan^®^, [Gd(OH_2_)(dtpa-bma)]. **c** Dotarem^®^, [Gd(OH_2_)(dota)]^−^. **d** Gadovist^®^, [Gd(OH_2_)(do3a-butrol)]. **e** Multihance^®^, [Gd(OH_2_)(bopta)]^2−^

The first contrast agent to incorporate gadolinium(III), [Gd(OH_2_)(dtpa)]^2−^ (Magnevist^®^), was synthesised in 1981 and approved by the FDA for clinical use in 1988^9^. Since then, a total of 11 GBCAs have been approved by the FDA, with the 5 most commonly used shown in Fig. 1^10^. Magnevist^®^, which has been administered globally almost 100 million times, dominates in the clinic^11^. However, issues with this widespread usage have presented themselves in recent years. Chief amongst these is the link between the rare, yet potentially fatal, condition nephrogenic systemic fibrosis (NSF) and the administration of a GBCA to patients with kidney failure, which first became apparent in 2006^12^. The deposition of gadolinium metal has been observed in the skin, heart and kidneys of patients with NSF and, more recently, the brains of patients with healthy kidney function^13–17^. In addition, Gd^3+^ ions have a similar ionic radius to Ca^2+^, enabling them to interfere with calcium-mediated signalling pathways—thus rendering them extremely toxic, with an LD_50_ of 0.2 mmol kg^−1^ observed in mice^18,19^. Concern over the safety of some GBCAs with acyclic ligands has resulted in the restriction of their administration by the European Medicines Agency (EMA) and triggered risk warnings from the U.S. Food and Drug Administration (FDA)^17,20,21^. The toxicity of GBCAs has been covered extensively elsewhere in the literature^22–24^, and strategies to increase complex stability through ligand design have often been motivated by the desire to reduce potential adverse effects on patients.

Due to the low sensitivity of MRI as an imaging technique, large quantities of a contrast agent, often on the gram scale, must be injected into the patient to obtain useful images. The ability to reduce the quantity of GBCAs required is highly desirable, especially when considering the toxicity problems discussed above. One way in which the amount of contrast agent required can be reduced is to enhance its relaxivity. Relaxivity is a measure of how water relaxation rate changes with concentration of a contrast agent, and high relaxivities are indicative of more effective agents^6,25,26^. Hydration state, i.e., the number of coordinated inner sphere water molecules, is a key contributor to a complex’s relaxivity, and increasing the hydration state is accompanied by a corresponding increase in relaxivity^4,25,26^. This can be seen from Eq. (1) ^27^, where *r* is relaxivity, *c* is concentration, *q* is hydration state, *T*_1m_ is longitudinal proton relaxation time and *τ*_m_ is water exchange lifetime, i.e., how long an inner sphere water molecule spends at the metal centre. All existing GBCAs that are used in the clinic are based on octadentate polyaminocarboxylate ligands. As trivalent gadolinium prefers a coordination number of 9, this leaves one available coordination site free for an inner sphere water molecule, allowing its relaxation rate to be enhanced, providing contrast.

1 $$r = \frac{{cq}}{{55.6}}\left( {\frac{1}{{T_{1{\mathrm{m}}} + \tau _{\mathrm{m}}}}} \right){.}$$

The relaxivity of a contrast agent can, therefore, be improved by increasing the hydration state of the complex. This can be achieved through a reduction in the number of coordination sites provided by the ligand as water molecules are generally displaced by more basic ligating species during coordination. Importantly though, this will typically lower the thermodynamic stability of the complex and render the metal ion more accessible to endogenous anions. This accessibility may lead to demetallation of gadolinium(III) complexes in vivo, causing further toxicity issues. Many in the field have tried to master the subtle interplay between maximising relaxivity through accessing higher hydration states and forfeiting thermodynamic stability or kinetic inertness. Several innovative strategies have been employed, the discussion of which form the basis of this review. Whilst several thorough and comprehensive assessments of GBCAs have been published, these have predominantly focussed on ligand design and other techniques to augment the relaxivity of gadolinium(III) complexes which could then, often hypothetically, be used as contrast agents^4,6,25,28^. This review article assesses approaches to ligand design that focus on achieving greater complex stability. Initially, we will establish what is meant by complex stability in terms of thermodynamics and kinetics, before discussing and assessing the effectiveness of modifications which have been made to the cyclic and acyclic ligand families used in GBCAs.

## Decisive stability factors

The high required dose of GBCAs for clinical use carries the risks of health problems associated with Gd^3+^ release, and thus requires the administration of very stable GBCAs. Gd^3+^ release by GBCAs can be characterised by thermodynamic stability and kinetic inertness^8,29–33^, which may be manipulated through ligand design^28^. Thermodynamic stability refers to the Gibbs free energy involved in the complexation reaction and is defined as stability constant log *K* (also called log *K*_GdL_, log *K*_therm_ and log *K*_st_). Kinetic inertness refers to complex dissociation rate and is mainly reported as *t*_1/2_, where *t*_1/2_ is defined as the time required for half of the GBCA’s dissociation. It should be noted that values for log *K* and *t*_1/2_ for a GBCA may differ slightly in the literature due to the differences between methodologies and experimental conditions used by different investigators.

The thermodynamic stability constant can be calculated by the equilibrium concentration of each component with the assumption that the ligand is fully deprotonated. There are several methods for the determination of log *K*. These include pH-potentiometry, spectrophotometry and proton relaxometry, which are respectively conducted by recording changes of pH value, absorbance/emission intensity or proton relaxivity of a solution containing equimolar amounts of metal ion and ligand as a function of added acid or base. Complexation of lanthanide(III) ions, including Gd^3+^, especially with macrocyclic ligands, is generally slow. Thus, the “out-of-cell” method is frequently used rather than direct titrations, i.e., a series of samples to span the pH range are prepared and allowed to equilibrate before measurements are made^34^. For complexes with a high log *K* value, the competition method can be used. This requires a competing reference ligand or metal ion. The hard acid Gd^3+^ favours basic donor atoms such as nitrogen and charged oxygen, which can be exploited to increase the log *K* of Gd^3+^ complexes. However, log *K* formally describes the reaction between a metal ion and a fully deprotonated ligand, with the latter only existing in solution at high pH values. At lower pH ranges, protons compete with Gd^3+^ to bind with the ligand. This additional consideration is taken into account by the conditional thermodynamic stability constant: log *K’* (also called log *K’*_GdL_, log *K*_cond_ and log *K*_eff_), a value measured at pH 7.4 using 1:1 ligand-to-metal ratio. Log *K*’ can be calculated from log *K* and ligand protonation constants. Less commonly, pGd (pGd = −log [Gd^3+^] at pH 7.4, freqcuently at a ligand to metal concentration ratio of 10:1 although a range have been used in the literature) has been suggested as a measure of complex conditional thermodynamic stability. Log *K*’ is, therefore, more useful than log *K* with respect to thermodynamic stability under certain conditions, i.e., physiological pH 7.4. Increased ligand basicity results in stronger proton binding and can result in a larger difference between log *K* and log *K’*.

The dissociation of GBCAs and release of Gd^3+^ in vivo is complicated. Competitive binding of Gd^3+^ by endogenous anions such as PO_4_^3−^ and CO_3_^2−^ and transmetallation by endogenous metal ions including Zn^2+^ and Cu^2+^ are two possible pathways towards demetallation. As precipitation of Gd^3+^ by competing biological anions is not a significant contributor to in vivo dissociation^35^, transmetallation represents the main pathway to induce Gd^3+^ release. Transmetallation of acyclic chelates was found to be associated with direct attack by endogenous Zn^2+^ (or Cu^2+^) ions, while for macrocyclic complexes, it occurs mainly through acid-assisted processes^36,37^. Thus, the dissociation rate of Gd^3+^ complexes is mainly measured in strong acids, often 0.1 M aqueous HCl, or evaluated by transmetallation experiments via spectrophotometry, HPLC and relaxometry. Laurent et al.^38,39^ have established a relaxometric method with a standard set of experimental conditions to assess the transmetallation of Gd^3+^ complexes. The method records the change of proton relaxation time in a pH 7 phosphate-buffered solution containing equimolar amounts of Gd^3+^ complex and Zn^2+^. As released Gd^3+^ precipitates as GdPO_4_, it can be assessed by measuring the increase in proton relaxation time. They used this method to evaluate all approved GBCAs and found that transmetallation is much quicker for acyclic chelates compared to macrocyclic species. Frenzel et al.^40^ investigated the release of Gd^3+^ in serum solutions, aiming to mimic physiological conditions (pH 7.4, 37 °C). The results showed that under these conditions, Gd^3+^ release by the approved GBCAs followed the order: non-ionic linear >ionic linear >macrocyclic. While physiological conditions were taken into account in measuring dissociation rate, the obtained values are more useful at allowing discrimination and comparison between GBCAs rather than truly reflecting their in vivo behaviour.

As discussed above, kinetic inertness indicates the rate of Gd^3+^ release, whilst thermodynamic stability describes how much Gd^3+^ is released at equilibrium under certain conditions. Since the rate at which equilibrium is reached for macrocyclic gadolinium(III) complexes in vivo is very slow and normally cannot be reached during their residence time, thermodynamic stability cannot accurately predict Gd^3+^ release for macrocyclic GBCAs. Thus, Tweddle et al.^41^ have concluded that thermodynamic stability alone is insufficient to predict the in vivo dissociation of macrocyclic chelates. Wedeking et al.^42^ have shown that ^153^Gd accumulation in mouse liver and bone tissue is proportional to the dissociation rate of several ^153^Gd chelates. Also, [Gd(OH_2_)(dota)]^−^ which is stable in both thermodynamic and kinetic terms, resulted in low in vivo deposition; while [Gd(OH_2_)(dtpa)]^2−^ with a high thermodynamic stability but low kinetic inertness, has had its administration restricted. The ligand tetra-glycine amide analogue of H_4_dota, H_4_(dota)(gly)_4_, forms a kinetically inert complex with Gd^3+^, and its analogous complex with Eu^3+^ has been confirmed as non-toxic in vivo, even at a much higher dosage (1.0 mmol kg^−1^) than clinically used—this is despite a much lower thermodynamic stability than [Gd(OH_2_)(dota)]^−^ ^8^. This combined information suggests that it is kinetic inertness rather than thermodynamic stability that appears to be the useful predictor of in vivo Gd^3+^ release from GBCAs. One recent striking finding is the observed gadolinium deposition in the brain for subjects with normal renal function and an intact blood–brain barrier^14,43–46^. Although uncertainty exists around the specific diseases and conditions associated with gadolinium deposition in the brain, it has been shown that Gd^3+^ in the brain is toxic^47–49^. Thus, maximising kinetic inertness should be a critical concern for those seeking to develop future GBCAs.

## Cyclic ligands for gadolinium(III)

One ligand is frequently referred to as the “gold standard” in trivalent gadolinium chelation: 1,4,7,10-tetraazacyclododecane-1,4,7,10-tetraacetic acid (H_4_dota). This ligand consists of the macrocycle cyclen, which is *N*-functionalised with four acetic acid pendant arms and is octadentate with four nitrogen and four oxygen donor atoms (Fig. 1c) and [Gd(OH_2_)(dota)]^−^ remains the most thermodynamically stable GBCA in clinical use^50^. Whilst there is some variation in the literature due to different measurement methodologies, the high stability constant for [Gd(OH_2_)(dota)]^−^ ranges from 24.0 to 27.0, and for the purposes of this review will be referred to as 25.6, the value determined by Moreau et al.^51^. Due to its excellent chelating abilities, H_4_dota has thus proven to be an attractive scaffold for modification.

**Fig. 2:**
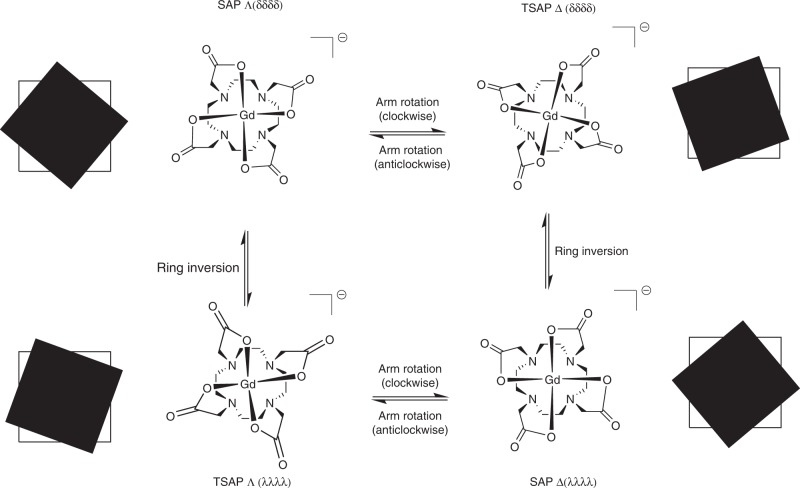
Illustration of the solution behaviour of Gd^3+^ complexes of H_4_dota. H_4_dota is an important cyclic ligand ubiquitous in clinical settings. There are four possible stereoisomers for [Gd(dota)]^−^ and interconversion between them is shown. The geometry can be visualised if the donor atoms are considered in planes, with the macrocyclic N4 donors forming one plane (white square), and the acetate O4 donors another (black square). Pairs of enantiomers are diagonally opposite one another. Individual atom charges and coordinated water molecules are omitted for clarity. (Adapted with permission from ref. ^55^)

To understand the logic behind some of the design strategies employed in this field, it is necessary to consider the solution behaviour of lanthanide(III) complexes of H_4_dota. There are known to be four different stereoisomers of [Ln(dota)]^−^ in solution, which arise from the orientation of the five-membered coordination metallacycles formed by ethylene bridges in the macrocycle (*λλλλ* or *δδδδ*) and the corresponding positions of the pendant arms (*Λ* or *Δ*)^26,52–55^. In geometric terms, the shapes adopted by these stereoisomers are described as square antiprismatic (SAP) or twisted SAP (TSAP), (Fig. 2)^26,54^. Each of these isomers may be characterised by the twist angle between the nitrogen and oxygen donor atom planes, *θ*^55^. This angle varies according to which Ln^3+^ centre the ligand is complexed to, but is typically around 40° for SAP structures and between −20° and −30° for TSAP geometries^2,55^. The two SAP and two TSAP isomers are enantiomeric pairs, and interconversion between them is possible on the nuclear magnetic resonance (NMR) timescale, leading to the broadening often seen in proton NMR spectra of lanthanide(III) complexes of H_4_dota^52^. Interconversion occurs through two mechanisms: rotation of the acetate arms or inversion of the macrocyclic ring^26,52–55^.

In order to maximise complex inertness, it is imperative that interconversion is minimised^26^. Chirality is widely exploited in catalysis and natural product synthesis to attain enantiomer formation, and similar principles may be applied to macrocyclic ligands. Introduction of chirality to the macrocycle itself and the pendant arms can render both interconversion mechanisms unfavourable and cause the complex to favour a particular geometry, thus reducing kinetic lability of the complex.

## Introduction of chirality into the macrocyclic ring

**Fig. 3:**
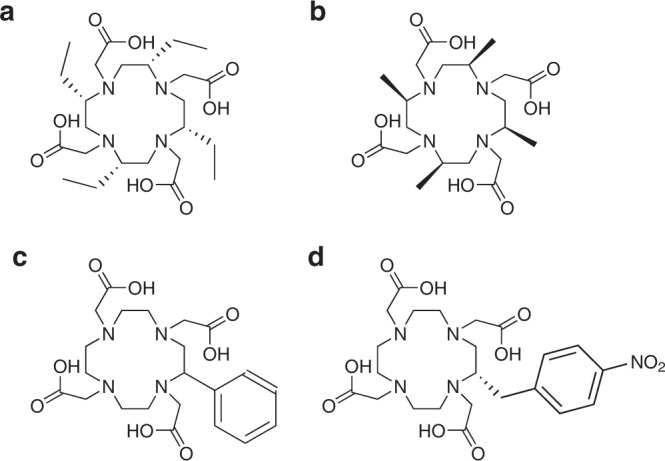
Ligands where chirality has been introduced into the N4-macrocyclic ring. Substitution on the ring has been exploited to render ring inversion unfavourable and thus encourage inertness of the resulting gadolinium complex. **a** Tetraethyl-substituted ligand H_4_Et_4_dota, all (*S*). **b** Tetramethyl-substituted ligand H_4_Me_4_dota, all (*R*). **c** Phenyl-substituted ligand H_4_Phdota. **d** Nitrobenzyl-substituted ligand H_4_nb-dota (*S*). Stereochemistry is added where known

Substitution on the macrocyclic ring itself has been exploited to render ring inversion unfavourable. Recently, Dai et al.^56^ published a comprehensive investigation of lanthanide(III) complexes of chiral chelators based on H_4_dota, and their solution kinetics. The group synthesised a series of H_4_dota analogues with substituents ranging in size from methyl to 4-aminobutyl, with the hypothesis that introducing chirality would lock ring conformation and aid ligand pre-organisation, resulting in greater complex inertness^56^. The synthesised gadolinium(III) complexes illustrated that increased steric bulk in the substituent resulted in a greater propensity for TSAP isomer formation, potentially due to steric clashes with the acetate arms rendering SAP geometry unfavourable. The SAP and TSAP isomers of the Gd^3+^ complex of the tetraethyl-substituted ligand (H_4_Et_4_dota, Fig. 3a) were separated and their kinetic inertness analysed^56^. Both isomers of the gadolinium(III) complex of H_4_Et_4_dota have been shown to survive for 7 days in the presence of 1000 equivalents of H_5_dtpa and exhibit no demetallation after 100 h at 50 °C in the presence of a 100-fold excess of ZnCl_2_^56^. The remarkable kinetic inertness is attributed to the influence of the chiral substituents, i.e., the introduction of stereochemistry enhances complex rigidity, leading to the restricted and non-interconvertible SAP and TSAP structures^56^. Ranganathan et al. reported lanthanide(III) complexes of tetra-methyl substituted H_4_dota (H_4_Me_4_dota, Fig. 3b). Here also, the introduction of chirality to the macrocycle was found to reduce the rate of isomer interconversion^57–59^.

One important consideration with this approach is that of over-rigidification, i.e., the steric bulk of the substituent dictating enantioselectivity is critical. Edlin et al. synthesised H_4_Phdota with the intention of increasing the energy barrier to ring inversion (Fig. 3c), as bulky cyclen substituents have been observed to introduce stereoselectivity in complexation^60^. However, the lanthanide(III) complexes were less thermodynamically stable and kinetically inert than those of H_4_dota^60^. The observation that large benzylic substituents can “lock” the conformation of the macrocycle into the *δδδδ* configuration forming SAP *Λ* (*δδδδ*) and TSAP *Δ* (*δδδδ*) isomers has been confirmed by the work of Payne and Woods with the synthesis of H_4_nb-dota, (Fig. 3d)^61,62^. The large steric bulk did not significantly impact the thermodynamic stability or kinetic inertness of the Gd^3+^ complex of H_4_nb-dota^62^. One possible reason could be the interconversion between two pairs of isomers (side or corner of the nitrobenzyl substituent on the macrocycle) which was not observed in the work of Dai et al.^56,61^. In employing this approach, it is clear that care must be taken when considering which substituents to introduce, i.e., enough steric bulk to confer enantioselectivity is critical and can favourably impact the inertness of gadolinium(III) complexes.

## Introduction of chirality into the pendant arm

**Fig. 4:**
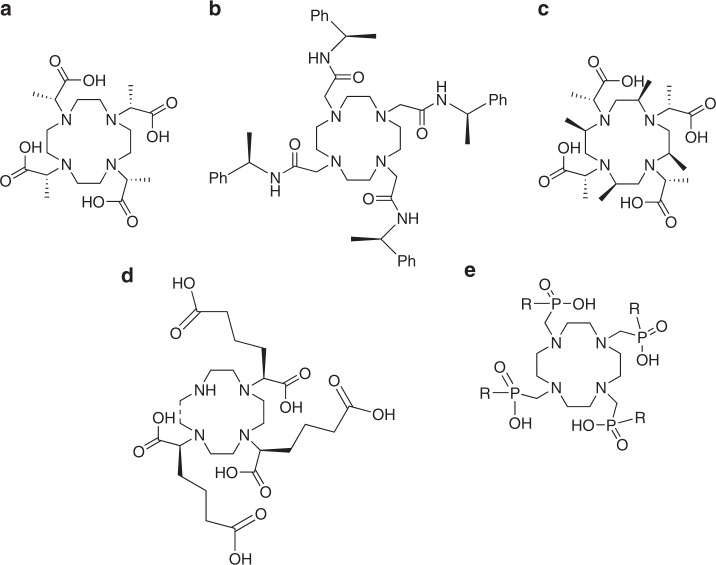
Ligands where chirality has been introduced into the pendant arm of the N4-macrocyclic ring. Substitution onto the arms can hinder isomer interconversion by preventing arm rotation and encouraging preference for a specific complex geometry. **a** H_4_dotma (all *R*), featuring a methyl group at the alpha position of the arm. **b** DotamPh (*R*), featuring a chiral substituent on each pendant arm. **c** H_4_Me_4_dotma (all *R*), where there is introduction of chirality into both the cyclen-N4 ring and the pendant acetate arms. **d** H_6_*R,R,R*-gado3a, with only three pendant arms, each of which is modified at the alpha position. **e** H_4_dotp^R^ (R = OH, H_8_dotp, R = H, H_4_dotp^H^, R = O-^*n*^Bu, H_4_dotpmb), where chirality is introduced via phosphinic or phosphonic acid pendant arms. Stereochemistry is added where known

Substitution onto the acetate arms of H_4_dota analogues has been investigated in an attempt to hinder isomer interconversion by arm rotation. (*R*)-2-[4,7,10-Tris-((*R*)-carboxyethyl)-1,4,7,10-tetraazacyclododecan-1-yl] propionic acid, H4dotma, which contains a methyl group at the α position of the arm, was originally reported by Brittain and Desreux in 1984, and has been extensively studied^63^. It was found that substitution on the arm introduces preference for one stereoisomer, (TSAP *Λ* (*λλλλ*) over SAP *Λ* (*δδδδ*) for the all (*R*) analogue) (Fig. 4a), with interconversion between the two stereoisomers possible by ring inversion, and that the lanthanide(III) complexes formed are conformationally rigid on the NMR timescale. This suggests that kinetic inertness may be enhanced by substitution at the α position of the acetate arm^59,63,64^. Despite this preference in geometry, it has been determined that the lanthanide(III) complexes of H_4_dotma are less thermodynamically stable than those of H_4_dota, albeit only slightly. Log *K* for the respective Gd^3+^ complexes have been reported as 23.6 and 24.7, respectively^64^. This is attributed by the authors to the less thermodynamic stable TSAP geometry adopted by H_4_dotma compared to the SAP geometry^64,65^. Another example of a chiral substituent on the pendant arm is that of 1,4,7,10-tetrakis-[(*R*)-1-(1-phenyl)ethylcarbamoylmethyl]-1,4,7,10-tetraazacyclododecane, dotamPh, the lanthanide(III) complexes of which adopt only SAP geometry in solution (Fig. 4b)^26,66,67^.

A preference for a specific complex geometry can also be enhanced through the introduction of chirality into both the cyclen ring and the acetate arms. This was achieved by Ranganathan et al.^57^, who synthesised the H_4_dotma derivative H_4_Me_4_dotma (Fig. 4c), which contains methyl groups on the macrocycle and at the *α* position. In solution, the free ligand is highly conformationally rigid, and this results in extremely slow exchange between isomers of the lanthanide(III) complexes. Subsequent investigations by Opina et al.^68^ have found that lanthanide(III) complexes of H_4_Me_4_dotma and analogous ligands are extremely rigid in solution, with very little interconversion between isomers taking place at ambient temperatures. [Gd(Me_4_dotma)]^−^ adopts an SAP geometry almost exclusively when all stereocentres are *S*, and the inverse is the case when they are all *R*^68^.

One innovative example of chirality being utilised for the enhancement of relaxivity and inertness comes from Messeri et al., who synthesised the ligand H_6_*R,R,R*-gado3a (Fig. 4d)^69^. This ligand possesses only three pendant arms, each of which is modified at the alpha position with the addition of a butanoic acid side chain. These additional carboxylic acid groups are deprotonated at physiological pH yet remain uncoordinated to the Gd(III) centre during complexation, likely due to steric hindrance. This renders the ligand heptadentate, allowing a hydration state of 2 to be accessed and a relaxivity value of 12.3 mM^−1^ s^−1^ to be obtained^69^. There is a high degree of kinetic inertness for [Gd(OH_2_)_2_(*R,R,R*-gado3a)]^3−^, which the authors report as being an order of magnitude greater than that of [Gd(OH_2_)(dtpa)]^−^ (*t*_1/2_ for [Gd(OH_2_)(dtpa)]^−^ is 4.5 h)^69^. However, when compared to the approved macrocyclic agents [Gd(OH_2_)(dota)]^−^ and [Gd(OH_2_)(hp-do3a)], both of which have a *t*_1/2_ greater than 83 h, [Gd(OH_2_)_2_(*R,R,R*-gado3a)] is still more kinetically labile^38,70^.

A slightly more unorthodox method to introduce chirality to macrocyclic ligands for Gd^3+^ utilises phosphinic acid or phosphonic acid monoester pendant arms, rather than acetic acid. This has been shown to increase the energy barrier to ring inversion to around 100 kJ mol^−1^ in [Ln(dotp)]^5−^ (Fig. 4e), a value higher than [Ln(dota)]^−^, which could theoretically enhance kinetic inertness by reducing the rate of isomer interconversion^71^. Extensive exploration of the effect of phosphonic acid substituents, [Ln(dotp^R^)]^*x*−^, on hydration state and relaxivity has been carried out, and this is discussed elsewhere^72,73^.

## Impact of ligand basicity and charge

**Fig. 5:**
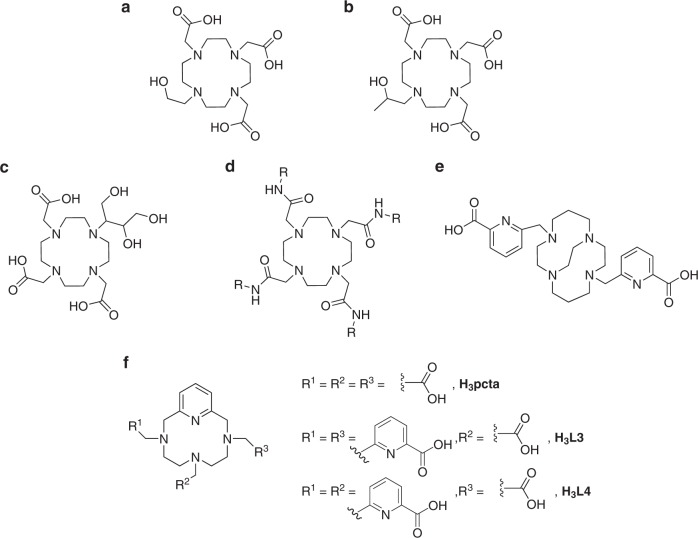
Ligands where substitution around the N4-macrocyclic ring affects physicochemistry. **a** H_3_hedo3a, featuring a methyl group within the alcoholic pendant arm. **b**, **c** H_3_hpdo3a and H_3_do3a-butrol both have three pendant negatively charged acetate arms. **d** H_4_-(dota)(gly)_4_ R = CH_3_, dtma, R = CH_2_COOH, comprising amido-functionalised N4-macrocyclic ligands with increased basicity. **e** H_2_cb-tedpa, featuring a reinforced N4-cyclam framework via an ethylene bridge. **f** H_3_pcta, H_3_**L3** and H_3_**L4**, featuring a heptadentate ligand incorporating pyridine directly into the ligand framework thus rigidifying and preorganising the ligand

A ligand’s basicity can be thought of as its affinity for protons and shown by the sum of protonation constant of each donor atoms in the ligand. Therefore, assumptions of how many protonation constants to include in the calculation need to be made. As early as 1990, Cacheris et al.^35^ showed that the more basic [Gd(OH_2_)(dtpa)]^2−^ demonstrated a higher thermodynamic stability than [Gd(OH_2_)(dtpa-bma)]. Later, one observation reported by Geraldes et al. from their work on 1, 4, 7, 10-tetraazacyclododecane 1, 4, 7, 10 tetrakis(methylenephosphonate) ligands, H_4_dotp^R^, was the reduced fluxionality of their lanthanide(III) complexes when compared to [Ln(dota)]^−^ species—something they attribute to the increased basicity of the phosphonate groups compared to acetate^71^. The idea of a linear relationship between thermodynamic stability and basicity for Gd^3+^ chelates was first reported by Kumar et al.^74^. The research investigated a series of 10-(2-hydroxyethyl)-1,4,7,10-tetraazacyclododecane 1,4,7-tetraacetate, H_3_hedo3a, derivatives with the introduction of a methyl group to the alcoholic pendant arm, the idea being to influence the basicity of the adjacent nitrogen (Fig. 5a). H_3_hedo3a was employed as an analogue of 1, 4, 7-tris (carboxymethyl)-1, 4, 7, 10-tetraazacyclododecane, H_3_do3a, replacing one carboxylate pendant arm with an alcohol group, thus decreasing its basicity. A linear correlation between log *K* and basicity was nicely displayed in this study, with the most basic, 10-(2-hydroxypropyl)-1,4,7,10-tetraazacyclododecane-1,4,7-triacetic acid, H_3_hpdo3a, showing the largest log *K*. Log *K’* is defined as log *K*/*a*_L_ (*a*_L_ is the sum of the stepwise protonation constants), thus log *K’* is neither proportional nor inversely proportional to ligand basicity but depends on specific structure. It was found that the ligand with intermediate basicity had the biggest log *K’*. Doble et al.^75^ reported a similar observation for the H_3_hopo-tam-based (hopo = hydropyridinonate; tam = 2,3-dihydroxyterephthalamide) gadolinium(III) chelates, as well as amongst aminoethyl-do3a derivatives^76^.

Greater basicity of the chelating ligand contributes to greater overall thermodynamic stability of the complex through stronger interactions with the Lewis acidic gadolinium(III) ion^77^. More stable complexes are also formed with ligands possessing negatively charged binding moieties, i.e., H_3_hpdo3a and 10-[2,3-dihydroxy-(1-hydroxymethyl)-propyl]-1,4,7,10-tetraazacyclododecane-1,4,7-triacetatic acid H_3_do3a-butrol (Fig. 5b, c) both have 3 acetate arms and their Gd^3+^ complexes exhibit log *K* values of 23.8 and 18.7, respectively^77,78^, compared to 25.6 for that of H_4_dota. The uncharged ligand dtma (Fig. 5d) forms a Gd^3+^ complex with a log *K* value of 12.8^79^. Other lanthanide(III) complexes have been formed with uncharged dota-tetraamide derivatives with *K* values up to 15 orders of magnitude lower than the corresponding dota^4−^ species^80^, indicating that a reduction in ligand charge is accompanied by a reduction in complex thermodynamic stability. This can be attributed to both enthalpic and entropic effects. The enthalpic contribution to the free energy change of complexation is comprised primarily of bond formation interactions and, as a result, the formation of stronger bonds gives rise to more stable lanthanide(III) complexes^81–83^.Whilst the dominant contribution to the reduced stability of tetraamide derivatives is enthalpic, entropy also has a role to play, i.e., more highly charged ligands are likely to be more hydrated in aqueous solution and complex formation will therefore require a greater degree of desolvation, liberating more water molecules^79^.

In kinetic terms, it is well-established that complex dissociation requires the displacement of a ligand donor atom. This can occur when a bond between a donor atom and a metal ion is weaker, perhaps due to a lower basicity of the ligand, or may be mediated by protonation of a donor atom^26,77^. One example addressing the impact of ligand basicity is a comparison study between H_4_dota and its phosphonate analogues 1, 4, 7, 10-tetraazacyclododecane-1, 4,7,10-tetrakis(methylenephosphonate), H_8_dotp, and the analogue H_4_dotpmb (Fig. 4)^84^. The strong basicity of H_8_dotp increased the thermodynamic stability constant of its Gd^3+^ complex to a value higher than [Gd(OH_2_)(dota)]^−^ ^85^, and also resulted in the presence of the protonated complex at physiological pH. Though this protonated species exhibited comparative relaxivity to [Gd(OH_2_)(dota)]^−^, the complex was found to have a strong affinity with bone and hydroxyapatite (by employing the radioisotopes [^153^Sm(dotp)]^5−^ and [^111^In(dotp)]^5−^)^86^, excluding it from use as a GBCA. The presence of non- or weakly coordinating groups susceptible to protonation can increase proton-mediated demetallation. A key example of this is seen with the ligands H_3_hpdo3a and H_3_do3a-butrol, which contain hydroxyl groups that are only weakly coordinated to the metal centre and these may be readily protonated^77^. Through intramolecular proton transfer mechanisms^87^, a proton can be transferred onto a cyclen nitrogen atom, resulting in dissociation^74,77^. Examples discussed in this section also show the linear relationship between ligand basicity and thermodynamic stability. However, in terms of kinetic inertness, increased ligand basicity results in an enhanced rate of proton-assisted dissociation.

## Introducing rigidity to complex ring systems

An interesting example of a different substitution technique for substitution onto the backbone of a macrocyclic ring comes from the work of Rodriguez-Rodriguez et al.^88^ in the form of the ligand 6,6′-((1,4,8,11-tetraazabicyclo[6.6.2]hexadecane-4,11-diyl)bis(methylene))dipicolinic acid, H_2_cb-tedpa, published in 2014. This ligand is based on a reinforced cyclam framework, with an ethylene bridge linking two nitrogen atoms which are *trans* to one another. Two picolinic acid moieties are bound to the other nitrogen atoms in the cyclam derivative (Fig. 5e)^88^.

The use of this structure as a framework may initially seem counter-intuitive, as it is known that the kinetic lability of trivalent lanthanide complexes of tetraazamacrocyclic ligands increases as the number of atoms in the ring increases, perhaps as the ligand becomes more flexible^89,90^. However, the introduction of a cross-linking structure appears to prevent this reduction in kinetic stability. The authors managed to successfully crystallise the europium(III) complex of H_2_cd-tedpa, which showed that the ligand completely envelops the metal ion and holds it in an extremely tight binding cavity^88^. This may be due to the enhanced rigidity of the ligand system. Recent work on a similar methylated cyclam system has suggested that this greatly enhanced kinetic inertness is due to the presence of the ethylene cross-bridge^88,91^. Another ligand family which has developed around this methodology is the pyclen family. Pyclen is closely related to the tetraaza macrocycle cyclen, but incorporates pyridine into the ligand backbone at one position^92,93^. In the mid-1990s, the ligand 3,6,9,15-tetraazabicyclo[9.3.1]pentadeca-1(15),11,13-triene-3,6,9-triacetic acid (H_3_pcta) was developed as a heptadentate chelator for lanthanide(III) ions (Fig. 5f)^92^. The heptadenticity of H_3_pcta enabled a hydration state of 2 to be accessed for the gadolinium(III) complex, with a corresponding increase in the complex’s relaxivity^92^. The introduction of pyridine into the ligand backbone was expected to rigidify this ligand, compensating for any potential reduction in thermodynamic stability or kinetic inertness associated with the reduction in denticity^93^. Indeed, the reported range of log *K* values for the [Gd(OH_2_)_2_(pcta)] complex was from 18.3 to 21.0, indicating comparable thermodynamic stability to the Gd^3+^ complexes of the octadentate ligands H_5_dtpa and H_4_cdta^93–95^. H_3_pcta also exhibited promising complex formation kinetics: the pyridine moiety introduces an additional degree of pre-organisation into the macrocyclic ring, resulting in formation rates of Ln^3+^ species that are an order of magnitude greater than those of H_4_dota^94^. However, other macrocyclic ligands such as H_4_dota and also the heptadentate H_3_do3a form gadolinium(III) complexes of greater thermodynamic stability than H_3_pcta^93^.

The initial promise of H_3_pcta has rendered it an attractive scaffold for further modification to enhance its stability and increase its suitability for potential in vivo applications. In 2003, Aime et al.^96^ reported the synthesis of a monosubstituted methylenephosphate derivative of H_3_pcta which formed a gadolinium(III) complex with a hydration state of 2. This complex exhibited a relaxivity value twice that of many clinically utilised contrast agents and an impressive log *K* value of 23.4, comparable to that of [Gd(OH_2_)(dota)]^−^^96^. However, it was only stable above pH 3^97^. More recently, Le Fur et al. have had success with the substitution of H_3_pcta’s acetic acid donor groups for picolinic acid^95,98^. Picolinic acid moieties are bidentate, and the synthesis of octadentate and nonadentate H_3_pcta derivatives incorporating this functionality have been reported. The disubstituted analogues reported in this work exhibit particularly impressive physical properties: both the symmetric (6-(carboxymethyl)-3,6,9,15-tetraazabicyclo[9.3.1]pentadecane1(15),11,13-triene-3,9-di(methylene)picolinic acid, referred to as H_3_**L3** by the authors) and asymmetrically substituted (9-(carboxymethyl)-3,6,9,15-tetraazabicyclo[9.3.1]pentadecane1(15),11,13-triene-3,6-di(methylene)picolinic acid, referred to as H_3_**L4**) variants (Fig. 5f) form Gd(III) complexes with pGd values greater than that of [Gd(OH_2_)(dota)]^−^ (e.g., 20.69, 21.83 and 19.21, respectively at a ligand to metal ratio of 10:1), and their reported log *K* values are extremely close (23.56 for [Gd(**L3**)] and 23.44 for [Gd(**L4**)])^95^. This greater thermodynamic stability almost certainly arises from the increased denticity of the ligands compared to H_3_pcta and increased rigidity over H_4_dota^95^. Despite the appeal of these ligands, they do have some shortcomings. The most promising, H_3_**L4**, forms a gadolinium(III) complex which has been found to have a hydration state of 0, limiting any potential application as a GBCA^95^. H_3_**L3** forms a gadolinium(III) complex with two solution structures, one of which is also *q* = 0, although the other exhibits a hydration state of 1. In addition, the half-life of [Gd(**L3**)] in 0.1 M HCl is only 14 h^95^. These low-hydration states could be a consequence of their nonadentate nature, or possibly the tight binding observed between the ligand and metal ion, one of the pitfalls in the search for greater thermodynamic stability^95^.

## Acyclic ligands for gadolinium(III)

2-[Bis[2-[bis(carboxymethyl)amino]ethyl]amino]acetic acid, also referred to as diethylenetraiminepentaacetic acid, (H_5_dtpa) was the ligand utilised in the first clinically approved GBCA —Magnevist®. Since then there has been enormous variation in the family of acyclic ligands used to chelate gadolinium(III), e.g., donor atoms have moved away from the archetypal mixed N and O systems seen in H_4_dota and H_5_dtpa to typically favour all oxygen donors. This has allowed reduced denticity to be explored to a much greater extent than with H_4_dota analogues.

## Exploiting basicity and oxophilicity

**Fig. 6:**
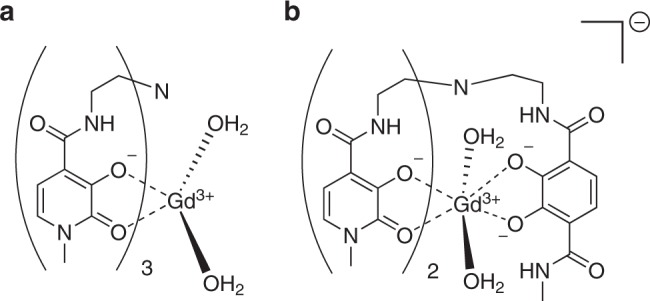
Exemplar chemical structures of Gd(III) complexes of the acyclic “hopo” ligands. Charged oxygen donors are a feature within the ligands: H_3_-tren-Me-3,2-hopo (complex **a**) and H_3_-tren-Me-3,2-hopotam (complex **b**). Enhanced stability is observed due to the significant oxophilicity of the Gd(III) ions

The basicity of donor atoms has a critical impact on thermodynamic stability and kinetic inertness of the resultant gadolinium(III) complex. This is exemplified by the development of hopo ligands, first reported in 1995. These exploit the greater basicity of charged oxygen over nitrogen and the known oxophilicity of the lanthanide(III) ions to form extremely stable complexes with gadolinium(III) centres^75,99–103^. Two of these ligands, H_3_-tren-Me-3,2-hopo and H_3_-tren-Me-3,2-hopotam (Fig. 6), form complexes with remarkably high pGd values of 20.3 and 20.1, respectively (ligand to metal concentration ratio of 10:1)^6,75,102,103^. These conditional stability constants compare with those of H_5_dtpa and H_4_dota under the same conditions (19.1 and 20.4, respectively), and are particularly noteworthy as the hopo ligands are hexadentate^6,26,75^. This reduced denticity allows a hydration state of 2 to be accessed, and a corresponding increase in relaxivity^6,26,102,103^.

The thermodynamic stability of these complexes is thought to arise from the strong bonds formed between the oxophilic Gd^3+^ centre and the Lewis basic oxygen donor atoms^6,75,104^. The origin of this stability is the basicity of the ligand. One caveat is that competition with protons is increasingly relevant as basicity increases, i.e., too high and the complex’s conditional stability constant actually begins to decrease, this is responsible for the higher pGd value observed for the [Gd(OH_2_)_2_(tren-Me-3,2-hopo)] complex than the chelate formed from the more basic H_3_-tren-Me-3,2-hopotam^75,100^. Raymond and Pierre hypothesised that the high selectivity of these ligands for the Gd^3+^ ion over the competitive cations Zn^2+^ and Ca^2+^, significantly more so than dtpa^5−^ and dota^4−^, could result in reduced demetallation if their Gd^3+^ complexes were to be utilised as GBCAs^75,100,101^. However, recent assessments have called the suitability of hopo ligands into question: the high-kinetic lability of the Gd^3+^ complexes, especially the fact that they are fully dissociated at pH values lower than 2, indicates that they may not be suitable in the clinic^101,105^. There is still limited in vivo kinetic data available for the Gd^3+^ complexes, and it is likely that, despite the initial promise and logical design strategies employed, other ligand systems will prove more appropriate for future development and translation of GBCAs^105^.

## Introduction of chirality to the ligand

**Fig. 7:**
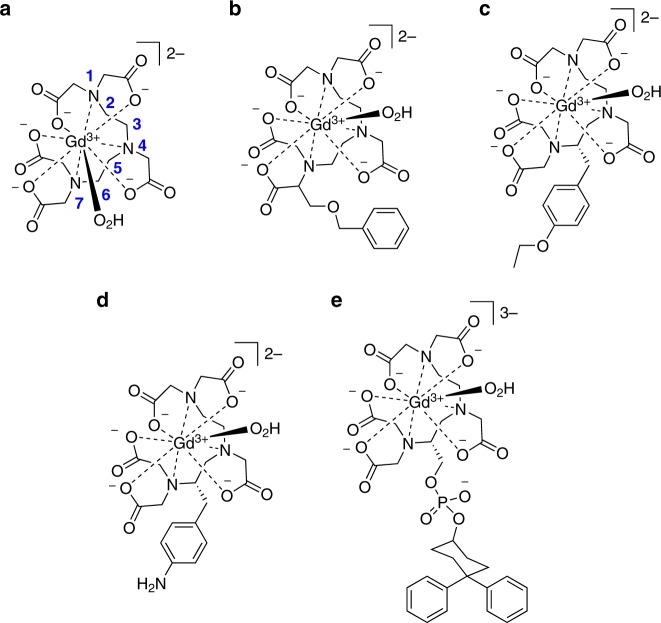
Acyclic ligands where chirality has been introduced into the ligand framework. **a** [Gd(OH_2_)(dtpa)]^2−^, where the central nitrogen atom N4 is chiral upon binding to Gd^3+^ but inversion is prohibited after complexation. **b** [Gd(OH_2_)(bopta)]^2−^, where substitution on the acetate ligand arm with a benzyl oxymethyl group facilitates chirality. **c** [Gd((*S*)-eobdtpa)]^2−^ and **d** [Gd((S)pNH_2_bz-dtpa)]^2−^, where chirality is introduced by bulky substitution on the ligand backbone. **e** The Gd(III) complex component of MS-325, where the steric bulk of the phosphodiester moiety encourages selectivity in isomer formation. Stereochemistry is added where known

The most favourable geometries adopted by nine-coordinate lanthanide(III) complexes are the monocapped SAP and the tricapped trigonal prismatic (TTP). [Ln(dota)]^−^ and derivatives adopt SAP or twisted SAP geometries, while [Ln(dtpa)]^2−^ and derivatives tend to adopt a distorted TTP geometry. In [Ln(dtpa)]^2−^, the dtpa^5−^ anion binds to the metal centre with three nitrogen atoms and five carboxylate oxygen atoms forming a distorted TTP^26,106,107^. Two enantiomers exist in solution, *λλ* and *δδ*, arising from the relative helicity of the C–C ethylene bond to the Ln(III)-N–N plane^107–109^. The central nitrogen atom N4 (Fig. 7a) is chiral upon binding to Ln^3+^, however, its inversion is prohibited after complexation. A relatively low-energy barrier to interconversion exists between enantiomers: typically around 50–60 kJ mol^−1^, and this can be observed through coalescence of ^1^H and ^13^C resonances in high-temperature NMR spectra of [Ln(dtpa)]^2−^ complexes^2,26,109–113^. Substitution on the diethylenetriamine backbone or the acetate arms introduces additional chirality to the ligand, potentially allowing the stereochemistry of the complex to be directed^26,106,114^. Introducing this preference in complex geometry raises the interconversion activation energy, enhancing kinetic stability of the complex^115^. Typically, groups which have a large steric bulk are employed in this strategy. One of the first examples was 4-carboxy-5,8,11-tris(carboxymethyl)-l-phenyl-2-oxa-5, 8, 11-triazatridecan-13-oic acid, H_5_bopta, in which one hydrogen atom on a terminal acetate group was replaced with a benzyloxymethyl group^106^. H_5_bopta can form gadolinium(III) complexes which exhibit a similar thermodynamic stability to those of H_5_dtpa, with a conditional stability constant of 18.4 under physiological conditions (Fig. 7b)^106,116^. Despite H_5_bopta’s initial promise, it was subsequently found to interact significantly with Zn^2+^ cations at physiological pH, which could result in demetallation in vivo^39^.

Examples of chirality introduced by bulky substitution on the backbone are *para*-aminobenzyl diethylenetriaminepentaacetic acid, H_5_pNH_2_-bzdtpa, and ethoxybenzyl diethylenetriaminepentaacetic acid, H_5_eobdtpa (Fig. 7c, d)^111,114,117^. In both ligands, with the (*S*)-absolute configuration of the introduced chiral centre C6, the central chiral nitrogen atom N4 after metal ion coordination and the ligand wrapping isomers, there are four possible diastereomers of the lanthanide(III) complexes^118^. In H_5_pNH_2_-bzdtpa, the *para*-aminobenzyl substituent gives a preference for the formation of the *Λ* enantiomer during complexation: the *Δ* isomer requires the bulky aminobenzyl substituent to adopt a position resulting in unfavourable steric clashes^114^. With H_5_eobdtpa, the ethoxybenzyl substituent was found to have minimal impact on ligand basicity, but there was a substantial increase in log *K* of the Gd^3+^ complex of the (*S*) enantiomer when compared to both H_5_dtpa and H_5_bopta^117^. It can be concluded that the introduction of chirality, particularly through substituents with an increased steric bulk, results in increased thermodynamic stability and kinetic inertness of H_5_dtpa analogues.

MS-325 is the trisodium salt of the gadolinium(III) complex of 2-(*R*)-[(4,4-diphenylcyclohexyl) phosphonooxymethyl] 2-[bis[2-[bis(carboxymethyl)amino]ethyl]amino]acetic acid, (Fig. 7e), where a large diphenyl cyclohexyl phosphodiester substituent was introduced within the ligand’s diethylenetriamine backbone^26,119,120^. The steric bulk of the phosphodiester moiety was found to invoke selectivity in isomer formation, preferentially adopting a TTP with *Λ* helicity, resulting in an increased kinetic inertness over the corresponding dtpa^5−^ complexes as the rate of isomer interconversion is reduced^110,111^. The large lipophilic substituent of MS-325 allows interaction with the blood protein human serum albumin (HSA)^111,121^, which enhances the lifetime of the species in vivo^111,119–121^. Despite its advantages, the high affinity of MS-325 for HSA renders it mostly suitable for blood-pool imaging and limits wider application.

## Rigidification of the ligand backbone

**Fig. 8:**
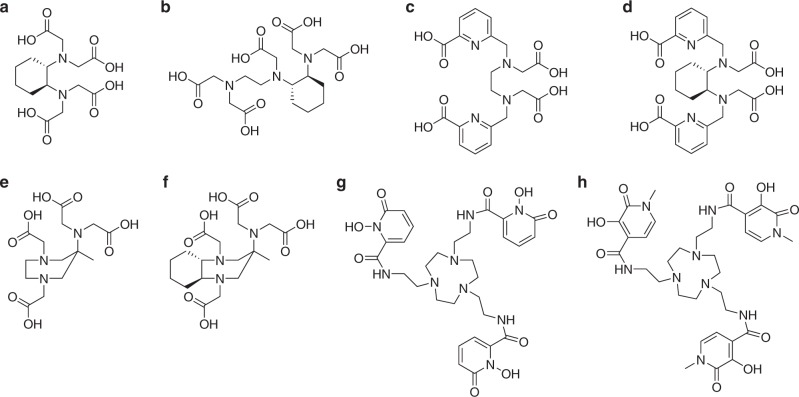
Acyclic ligands where rigidification has been introduced into the ligand framework. **a** H_4_cdta, featuring rigidification by a cyclohexyl motif. **b** H_5_chxdtpa, where a cyclohexyl derivative has also been included into the ligand backbone. **c** H_4_octapa, featuring two picolinic acid binding motifs giving octadentate ligand binding. **d** H_4_chxoctapa, another octadentate ligand again featuring cyclohexyl substitution for rigidification. **e** H_4_aazta, a heptadentate ligand based on an unusual seven-membered ring. **f** H_4_cyaazta, based on the seven-membered ring motif with additional use of a cyclohexyl group. **g**, **h** H_3_tacn-1,2-hopo and H_3_tacn-3,2-hopo, where a small N3-azamacrocycle motif is introduced to aid rigidification but not to aid in the Gd(III) complexation. Stereochemistry is added where known

Demetallation can occur when the staggered conformation of the diethylenetriamine backbone of H_5_dtpa is inverted. One strategy that has been employed to limit this process is rigidification. The cyclohexyl motif has been extensively used to rigidify acyclic ligands. Early work focused on H_4_cdta (Fig. 8a), the cyclohexyl derivative of ethylenediamine tetraacetic acid, H_4_edta. This ligand was found to have restricted flexibility and an extremely rigid coordination cage^122^. Due to this rigidity, cdta^4−^ complexes are more resistant to demetallation, with a high activation barrier to dissociation, especially when compared to many other acyclic ligands^122^. However, H_4_cdta also exhibits slow complex formation kinetics, with its lanthanide(III) complexes exhibiting smaller formation rate constants than those of the macrocycle H_4_dota^123^. The cyclohexyl variant of H_5_dtpa, H_5_chxdtpa (Fig. 8b), has also been synthesised, and forms lanthanide(III) complexes with greater thermodynamic stability than its parent ligand^124^. This strategy has been employed by other groups to increase the kinetic inertness of gadolinium(III) complexes. One notable example is that of 6,6′-[(ethane-1,2-diylbis((carboxymethyl)azanediyl)bis(methylene)]dipicolinic acid (H_4_octapa) (Fig. 8c). This octadentate ligand, which utilises two picolinic acid binding motifs, was complexed to Gd^3+^ by Kálmán et al.^125^, who subsequently investigated its solution equilibria. The [Gd(OH_2_)(octapa)]^−^ complex was found to have a log *K* value of 20.39, showing remarkable thermodynamic stability for an acyclic ligand complex^125,126^. However, the kinetic inertness of [Gd(OH_2_)(octapa)]^−^ was found to be unacceptably low for consideration as a viable GBCA, with a dissociation rate 20 times higher than [Gd(OH_2_)(dtpa)]^2−^ in the presence of competitive Cu^2+^ ions^125^. Tircsó et al.^126^ substituted the ethylenediamine backbone for the *trans*-1,2-diaminocyclohexane moiety discussed above to create the cyclohexyl derivative H_4_chxoctapa (Fig. 8d). The corresponding gadolinium(III) complex was found to have a marginally increased thermodynamic stability, exhibited by its log *K* value of 20.68 and pGd of 19.66 (ligand to metal ratio of 10:1)^126^. These values compare to complexes of many macrocyclic ligands in clinical use^126^. Of particular note was the enhancement in half-life of [Gd(OH_2_)(chxoctapa)]^−^ at physiological pH, being 1.49 × 10^5^ h. Under the same conditions, *t*_1/2_ for [Gd(OH_2_)(octapa)]^−^ was 0.15 h, and that for [Gd(OH_2_)(dtpa)]^2−^ 202 h^126^. This dramatic increase in kinetic inertness is attributed to the increased ligand rigidity, brought about through the cyclohexyl moiety in the backbone. This same technique has also found success in species with hydration states greater than 1, as reported for H_4_CyPic3A by Gale et al.^127^.

6-Amino-6-methylperhydro-1,4- diazepinetetraacetic acid, H_4_aazta, first synthesised by Aime et al. in 2004, is a heptadentate ligand based on an unusual 7-membered ring (Fig. 8e). The gadolinium(III) complex of H_4_aazta exhibited high thermodynamic stability, despite its reduced denticity compared to H_5_dtpa, its log *K* value being 19.26. The complex did not show any demetallation at pH 2, and was also highly selective for Gd^3+^ over many endogenous cations, i.e., no transmetallation of [Gd(OH_2_)_2_(aazta)]^−^ was observed in the presence of a tenfold excess of ZnCl_2_, CaCl_2_ or MnCl_2_. However, the complex is more labile than macrocyclic GBCAs^128^. [Gd(OH_2_)_2_(aazta)]^−^ has a hydration number of 2, which comes with a corresponding increase in relaxivity^125,129^. Introduction of the cyclohexyl motif proved interesting and was achieved by Vágner et al.^128^, who synthesised the ligand *trans*-3-amino-3-methyldecahydro-1*H*-1,5-benzodiazepine-*N*,*N*′,*N*′′,*N*′′ tetraacetic acid, H_4_cyaazta (Fig. 8f). The cyclohexyl ring results in a reduction in the thermodynamic stability of [Gd(OH_2_)_2_(cyaazta)]^−^ when compared to [Gd(OH_2_)_2_(aazta)]^−^ (log *K* = 18.26 and 20.24, respectively), as it rigidifies the coordination cage and prevents optimal accommodation of the metal ion^128^. The Gd(III) complex has a calculated half-life at pH 7.4 of 91 years, two orders of magnitude greater than [Gd(OH_2_)_2_(aazta)]^−^ itself, the highest reported for an acyclic *q* = 2 gadolinium(III) complex^128^. As discussed earlier, it is the inertness of a Gd^3+^ complex which can determine its potential in vivo toxicity. Although [Gd(OH_2_)_2_(cyaazta)]^−^ exhibits a long half-life for a complex of an acyclic, heptadentate ligand with a hydration state of 2, this pales in comparison with that of [Gd(OH_2_)(dota)]^−^, which has a half-life more than 3000 times greater under similar conditions, and other macrocyclic species, which would likely hinder the former’s application as a GBCA.

Another example of a ligand family with a ring system incorporated into its framework is the tacn-hopo series (Fig. 8g, h). These ligands, first reported in 2007 by Werner et al.^99^, are based on the macrocycle 1,4,7-triazacyclononane (tacn) and the hopo ligands discussed above^26^. The combination of rigidity imparted by the macrocyclic ring and the use of hard oxygen donors results in tacn-hopo ligands forming very thermodynamically stable complexes with gadolinium(III), with a pGd value of 18.7 (ligand to metal concentration ratio of 10:1) reported for the Gd^3+^ complex of H_3_-tacn-3,2-hopo^99^. Whilst this is lower than many macrocyclic GBCAs in clinical use, it is particularly impressive as the hexadentate ligand results in a complex hydration state of 3^26,99^. This results in the high relaxivity values for the Gd^3+^ complexes of H_3_-tacn-3,2-hopo and H_3_-tacn-1,2-hopo of 13.1 and 12.5 mM^−1^s^−1^ respectively, among the highest known for low weight mononuclear gadolinium(III) complexes^99^.

## Perspective

The range of techniques and ideas which have emerged from the pursuit of more stable GBCAs is a testament to the ingenuity and creativity of coordination chemists. Acyclic ligands, which were at the forefront of the field at its inception in the 1980s and historically have dominated clinical usage, have been the subject of many of these developments, ranging from the alteration of ligand basicity and the nature of the donor atoms to the manipulation of isomer ratios via chiral backbone substituents. Amongst these techniques, perhaps the most successful has been rigidification of the ligand backbone through the introduction of a cyclohexyl motif, as seen with the derivatives H_4_chxoctapa and H_4_cyaazta, the Gd(III) complexes of which are far more kinetically inert than Magnevist^®^. However, one critical factor prevents these ligands from spawning the next generation of GBCAs: they are just not inert enough. The thermodynamic stabilities of many acyclic ligands are indeed comparable to their macrocyclic counterparts but although stable complex formation is vital, this alone cannot predict the extent of Gd^3+^ release in vivo. Kinetic inertness is of paramount importance in the development of GBCAs, hence the recent action by both the FDA and EMA restricting the application of contrast agents based on acyclic species. For this reason, despite logical and thoughtful design processes applied to acyclic ligands by countless researchers over the decades, macrocyclic chelators are almost certain to be the basis of future clinical developments in this field.

Amongst the myriad of macrocyclic ligands discussed in this review, some show more promise than others, and all require development in the future. Alteration of ligand basicity has proven popular, but this is limited in practical use, i.e., increased basicity results in more rapid proton-mediated dissociation and can lead to complicated interactions in biological systems. Addition of chirality to the cyclen backbone and pendant arms of H_4_dota to enhance complex geometry preferences is a widely used technique, but one that must be approached with caution i.e. the new substituent must be large enough to invoke the necessary isomer selectivity, but not so bulky that the ligand is over-rigidified and cannot bind to the metal ion centre tightly enough. Thus, an optimum degree of substitution and size exists and will doubtless be found by skilled chemists in future. Ligand rigidification without the introduction of chirality has also exhibited potential, especially considering the remarkable inertness of some Gd(III) complexes formed with nonadentate pyclen derivatives and cross-bridged cyclam species, which also utilise the enhanced denticity of picolinic acids over traditional acetate arms. These scaffolds, which have only come to light in the past five years or so, may well be significant as work in the field of GBCAs progresses, and hopefully moves safer and more efficient GBCAs towards the clinic. The role of the coordination chemist will be instrumental to this translation—of course, working alongside biologists, imaging scientists and clinicians.
